# Magneto-optical plasmonic heterostructure with ultranarrow resonance for sensing applications

**DOI:** 10.1038/srep28077

**Published:** 2016-06-16

**Authors:** Daria O. Ignatyeva, Grigory A. Knyazev, Pavel O. Kapralov, Giovanni Dietler, Sergey K. Sekatskii, Vladimir I. Belotelov

**Affiliations:** 1 Lomonosov Moscow State University, Faculty of Physics, Leninskie Gory, 119991, Moscow, Russia; 2 Russian Quantum Center, Novaya str., 143025, Skolkovo, Moscow, Russia; 3 École Polytechnique Fédérale de Lausanne, Laboratoire de Physique de la Matière Vivante, IPHYS, CH-1015, Lausanne, Switzerland

## Abstract

Currently, sensors invade into our everyday life to bring higher life standards, excellent medical diagnostic and efficient security. Plasmonic biosensors demonstrate an outstanding performance ranking themselves among best candidates for different applications. However, their sensitivity is still limited that prevents further expansion. Here we present a novel concept of magnetoplasmonic sensor with ultranarrow resonances and high sensitivity. Our approach is based on the combination of a specially designed one-dimensional photonic crystal and a ferromagnetic layer to realize ultralong-range propagating magnetoplasmons and to detect alteration of the environment refractive index via observation of the modifications in the Transversal Magnetooptical Kerr Effect spectrum. The fabrication of such a structure is relatively easy in comparison with e.g. nanopatterned samples. The fabricated heterostructure shows extremely sharp (angular width of 0.06°) surface plasmon resonance and even sharper magnetoplasmonic resonance (angular width is 0.02°). It corresponds to the propagation length as large as 106 μm which is record for magnetoplasmons and promising for magneto-optical interferometry and plasmonic circuitry as well as magnetic field sensing. The magnitude of the Kerr effect of 11% is achieved which allows for detection limit of 1∙10^−6^. The prospects of further increase of the sensitivity of this approach are discussed.

The operating principle of the surface plasmon resonance (SPR) sensor is based on the registration of the resonance associated with the excitation of surface plasmon-polaritons (for brevity, below we will use simply the word plasmons here) which are electromagnetic waves propagating along the metal–dielectric interface. The spectral and angular positions of this resonance depend strongly on optical properties of the surrounding medium, including its refractive index and gyrotropy. Typically, such sensors exploit SPR in gold films and have the (bulk) refractive index resolution limit at the level of 10^−5^ refractive index units (RIU). By careful design and sophisticated signal processing, in certain cases the latter can be improved up to 10^−6^ RIU or even 2∙10^−7^ RIU, see^1^,^2^,^3^,^4^,^5^ and especially Fig. 4 in ref. 2. However, the improvement of the sensor sensitivity (which is the derivative of the acquired measurement with respect to the analyzed refractive index change) and resolution limit (as well as reliability (reproducibility) and relative ease to work with) remains essential for broadening of the application area since the direct detection of the lower concentrations and/or the detection of smaller molecules is an important problem of various biological, chemical and medical investigations, and a few directions of possible improvements were attempted.

Nanoplasmonic structures, such as gratings, nanoholes, nanoparticles, etc open a way for further SPR-sensor development^6^,^7^,^8^,^9^. Localized surface plasmon resonances can be excited in such structures so that the electromagnetic field is concentrated in a very small area near the nanoparticle that makes localized surface plasmon resonance very sensitive to the refractive index of the surrounding media and, as a result, some localized surface plasmon resonance-based sensors showed very high sensitivity. However, the technology of the fabrication of such nanoplasmonic structures is rather complicated for the commercial production and use of nanostructures for sensing is more difficult from the technological point of view compared with the conventional smooth layered plasmonic systems.

Progress with sensing was pushed forward also by excitation of the ultra-long propagating surface plasmons, see e.g.^10^,^11^,^12^,^13^,^14^,^15^. Indeed, the sensitivity is proportional to the derivative of the angular spectrum of the structure and increases for resonances of higher quality factor. At the same time the quality factor of the resonance is determined by the ratio of the real to imaginary parts of the surface plasmon polariton propagation constant and gets highest values for the ultra-long-range propagating modes.

The ultra-long-range propagation was first demonstrated in symmetrical multilayer structures when thin (8–20 nm) metal layer is “sandwiched” between two identical dielectrics^10^,^14^. Later it was recognized and practically demonstrated that to realize such ultra-long propagating plasmons, one of this dielectric layers can be replaced with a properly designed one dimensional photonic crystal (transparent multilayer coating)^11^,^12^,^13^,^15^. The surface plasmons in such structures are confined to the metal film due to the total internal reflection from the analyzed dielectric on the one side and due to the photonic crystal bandgap on the other. The parameters of the photonic crystal are tuned to match its effective impedance to the impedance of the dielectric below the metallic thin film so that the structure becomes quasi-symmetric and supports the long-range propagating surface plasmon polariton modes. This proved to be very important to work with low refractive indices media (gases and liquids) that usually carry biological or chemical objects to be detected^11^,^12^,^15^.

Another way for improvement is utilization of the magnetic layers in the plasmonic structure to measure a magnetooptical response instead of the reflection one^16^,^17^,^18^,^19^,^20^,^21^. In this respect, the transverse magnetooptical Kerr effect (TMOKE) is usually considered. It causes the difference between light reflectances of the structure magnetized in two opposite directions in transversal configuration, and for quantitative analysis such a difference is usually normalized on the reflectance in the non-magnetized case. Magnetic response of the heterostructure is greatly enhanced near the SPR^22^,^23^,^24^,^25^,^26^,^27^,^28^,^29^,^30^,^31^; moreover, the TMOKE resonance is narrower than the optical one which is a key point for the sensitivity and detection limit improvements. The sensing process of the magnetooptical sensor can be realized both via the spectral measurement of the shift of the TMOKE resonance position or via the measurement of the TMOKE value variation for the fixed near-resonant angle and wavelength.

The plasmonic structures of magnetic SPR-sensors contain ferromagnetic metals such as iron or cobalt, which, on the one hand, provide rather high magnetooptical response with a magnitude of several percentages, but, on the other hand, also significantly broaden the SPR due to high optical losses. Consequently, ferromagnetic metals are used in combination with noble ones to find a balance between the increase of the magnetooptical effects and the decrease of the resonance quality factors. Throughout the paper, when speaking about the quality factor we mean the ratio of the full width at half maximum (FWHM) of the resonance to its central wavelength. The recently demonstrated MO sensors^16^,^17^,^18^,^19^,^20^,^21^ still have rather poor quality factor of both optical and magnetooptical resonances, but nevertheless they often show the resolution limit several times higher compared to the conventional SPR ones.

In this work we present the magnetooptical heterostructure based on thin ferromagnetic cobalt layer, gold layer and a specially designed photonic crystal supporting ultralong surface plasmon propagation. The magneto-optical response is introduced by cobalt, while the quality factor of the plasmon resonance is determined by the photonic crystal and thicknesses of the gold and cobalt layers. Thus both aforementioned improvements, viz. the high quality factor of the plasmon resonance, and magnetooptical measurements instead of classical optical (SPR-influenced reflectance) ones are used simultaneously and synergetically. This allows us to obtain the unprecedentedly narrow angular TMOKE resonance width of 0.02° and quality factor of 2000 paving the way for ultrasensitive measurements with such sensor. On the other hand, the observed narrow SPR corresponds to excitation of ultra long-range magnetoplasmon decaying at a distance of about 100 μm.

## Results

### Ultra-narrow plasmon and magnetooptical resonances

**Figure 1:**
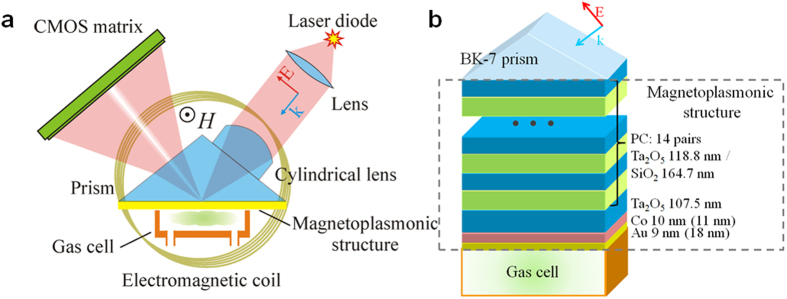
Magnetoplasmonic sensor scheme. Scheme of the experimental setup (**a**) and sensing magnetoplasmonic heterostructure (**b**).

The sensor scheme is presented in Fig. 1a (see Methods section for details). It comprises the laser diode, the magnetophotonic plasmonic heterostructure placed inside the electromagnet and contacting the cell with analyte gas, and CMOS matrix. Laser light is focused onto the magnetoplasmonic heterostructure surface in such a manner that the central angle of incidence of roughly the 3°–diverged beam corresponds to the resonant surface plasmon excitation angle in Kretschmann scheme. Angular interrogation is used: light reflected from the heterostructure surface forms a divergent beam which without any focusing is detected by the CMOS matrix. For the magnetooptical measurements, the sensor heterostructure was magnetized in the transversal configuration by the electromagnet. The angular spectra of the TMOKE thus were obtained as the difference between the two spectra of the reflectance measured with the opposite magnetization directions and normalized on the sum of these two spectra.

We carried out experiments for two samples of the magnetoplasmonic heterostructures: sample with 18-nm-thick Au layer (sample-2) which proved to be sufficient for a long time protection of 11-nm-thick Co layer from oxidation, and sample with 9-nm-thick Au film (sample-1). Such a thickness of gold layer makes the SPR resonance extremely sharp although it provides less long-term protection of 10-nm-thick Co layer to oxidation. The exact values of the metallic layer widths were obtained according to optical measurements of the transmittance spectra and fitting of the experimental data with spectra acquired by numerical simulations (Supplementary Fig. 1S). Based on these values reflectance and TMOKE spectra were calculated to find proper observation wavelengths and incidence angles (Supplementary Fig. 2S). The parameters of the sample layer widths and permittivities used for numerical simulations are described in Methods.

**Figure 2:**
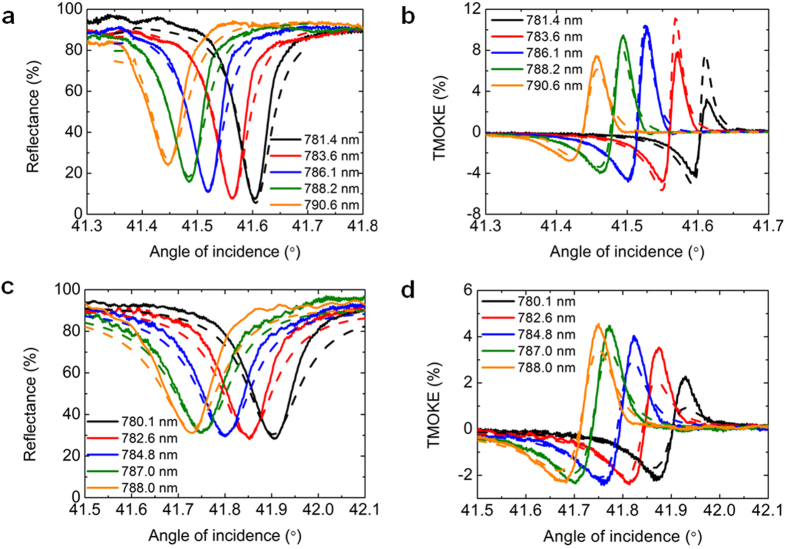
Ultra-narrow reflectance and magnetooptical resonances in the designed structure. Reflectance (**a,c**) and TMOKE (**b,d**) spectra of the sample-1 (**a,b**) and the sample-2 (**c,d**) at different wavelengths. Solid lines correspond to experimentally obtained spectra while dashed lines demonstrate the calculation results. Detailed parameters of the structures are described in Methods.

The measured reflectance (Fig. 2a) and TMOKE (Fig. 2b,d) angular spectra for different wavelengths confirm the excitation of the ultalong-propagating plasmonic mode in both samples. Numerical simulations well describe the experimental spectra. The experimental width of the SPR in the sample-1 (Fig. 2a) is up to 0.06° which gives the quality factor of 700 at the wavelength of 781.4 nm while the sample-2 (Fig. 2c) has 0.1° SPR width and its quality factor is 400 at the wavelength of 780.1 nm. Due to such a difference in the quality factors, the values of the TMOKE for these samples differ by more than 2 times. Also it is clearly seen that the magneto-optical resonance is sharper than the optical one: its angular width is 0.02° for the sample-1 at the wavelength of 786.1 nm and 0.04° for the sample-2 at the wavelength of 788 nm. This makes the quality factor of the magnetoplasmonic resonance to be as high as 2000 and 1000, correspondingly.

As SPR shape and spectral position are determined by the mode propagation constant, one can approximately estimate complex value of the propagation constant from the reflectivity spectra. Real part of the propagation constant Re[*β*] corresponds to the SPR angle *θ*_*SPR*_: Re[*β*] *=* *k*_*0*_*n* sin (*θ*_*SPR*_), where *n* is refractive index of the prism. As the shape of the SPR resonance near the dip can be approximately described by Lorentzian shape the imaginary part Im[*β*] of the propagation constant is determined by the resonance width Δ*θ* as Im[*β*] = 0.5 *k*_*0*_*n*Δ*θ* cos(*θ*_*SPR*_). Therefore ultra-narrow width of the SPR resonance demonstrated in our experiments points out that here we excite ultra-long propagating magnetoplasmons with propagation length of 106 μm. For comparison, at the same wavelength the propagation lengths of the magnetoplasmons on cobalt-air interface is only 4 μm and the propagation length at gold-air interface is 40 μm. Such long propagation distance of the surface plasmon polariton is unique for magnetoplasmonic structures, especially with lossy ferromagnetic metals. This makes the considered structure valuable not only for SPR sensing but also for magnetoplasmonic interferometry where propagation distances for surface plasmons typically do not exceed 20 μm (see, for example^26^).

TMOKE depends both on the derivative of the reflectance spectrum and its absolute value, hence the wavelength and angle corresponding to the maximal TMOKE differ from the wavelength and angle corresponding to minimal reflectance value (Fig. 2). The wavelengths of maximal TMOKE (786.1 nm for the sample-1 and 788 nm for the sample-2) have been used as the operational ones.

Position of the TMOKE resonance is determined by the position of the SPR, consequently resonances in both optical and magnetooptical spectra experience equal shifts due to the variation of the operating wavelengths or analyte substances. However magnetooptics provides significant improvement for sensing since by tuning the parameters of the magnetoplasmonic structure one can obtain higher derivative of the TMOKE resonance with respect to incidence angle in comparison with the SPR. Sharper TMOKE resonance allows for more precise measurements of the TMOKE resonance position and shift compared to the reflectance resonance.

### Gas sensing

To test the structure for sensing, we analyzed the reflectance and TMOKE spectral changes occurring due to the variations of gases filling the gas cell and contacting with the sensing heterostructure surface (see scheme in Fig. 1a). We used air with the refractive index *n* = 1.000292, helium with *n* = 1.000035, and butane, which refractive index was measured to be equal *n* = 1.00076 (see below), during the sensing process as analyte gases.

**Figure 3:**
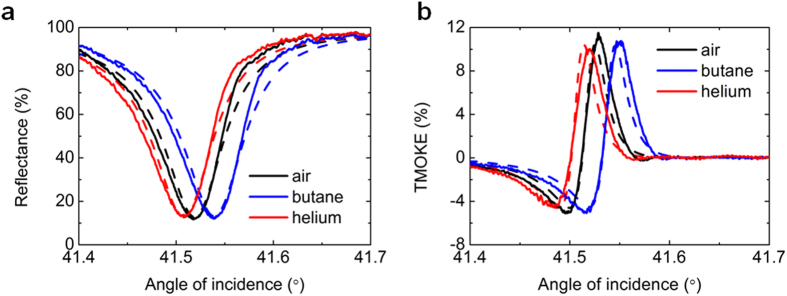
Reflectance and magnetooptical spectral variations during the gas sensing process. (**a**) Reflectance and (**b**) TMOKE spectra of the sample-1 measured for air, helium and butane as analyte gases. Solid lines correspond to experimentally obtained spectra while dashed lines demonstrate the calculation results. Detailed parameters of the structures are described in Methods.

Figure 3 shows how the spectra of the SPR and TMOKE of the sample-1 are modified when the gas cell is filled with different analyte gases. The experimental data obtained for helium and air show that the sensitivity of the resonance position to the refractive index changes is 43.2°/RIU for the sample-1 and 42.2°/RIU for the sample-2, respectively. Taking into account the TMOKE resonance FWHM which is 0.02° for the sample-1 and 0.04° for the sample-2 one may estimate another important characteristic of the sensor – its figure of merit. Figure of merit is determined as the ratio of the sensitivity to the width of the corresponding resonance and equals to 2200 and 1050 for two samples, respectively. At the fixed incidence angle the variation of the reflectance is 6 ∙ 10^4^%/RIU in the sample-1 (at 41.52°) and 2 ∙ 10^4^%/RIU in the sample-2 (at 41.72°). On the other hand, TMOKE value variation is 4 ∙ 10^4^%/RIU in the sample-1 (at 41.52°) and 4 ∙ 10^3^%/RIU in sample-2 (at 41.78°). The TMOKE variation for the sample-1 exceeds previously reported values by more than one order of magnitude, as can be estimated from the published data^19^,^20^. It results from the ultra-narrow SPR of the designed structure. Accordingly, the refractive index of butane gas (blue curves in Fig. 3) has been measured as equal to *n* = 1.00076.

## Discussion

In the current paper we will not discuss in details the sensitivity and detection limit of the “standard” SPR measurements with our heterostructure. First, these general questions and different related with them aspects have been profoundly discussed in the literature, see e.g.^1^,^2^,^32^,^33^, and, what is even more relevant and important now, here the situation is quite similar to the earlier presented sensors based on photonic crystal – supported ultralong propagating plasmons in thin metal layers^11^,^12^,^13^,^14^. All what has been discussed in the aforementioned papers remain relevant with only minor modifications, so it suffices to say that the sensitivity and detection limit of our heterostructure, provided optical SPR-related reflectance measurements are at stake, are roughly the same as was reported there (just “case-to-case” compare our current data, viz. an angular width of the plasmon resonance peak of 0.06° and reflection dip value around 90%, with the corresponding data from^11^,^12^,^13^,^14^). Instead, let us concentrate on the magnetooptical measurements where the question of sensitivity and detection limit is much less understood and remains debated.

Certainly, the magnitude of the magnetooptical signal is always lower than that pertinent for the SPR – based reflection (in our particular case, correspondingly ca. 16% and 90%), but direct application of the broadly accepted formula for the SPR sensor sensitivity, 
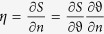
, where *S* is the signal at question, *n* is the measured refractive index and 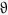 is the plasmon resonance angle, is inappropriate for the comparison of different approaches due to the different natures of the measurements; as this was many times stated, the corresponding signal-to-noise ratio should be properly taken into account. Typical baseline noise in this and other magnetoplasmonic sensing experiments (sf. e.g.^21^) is 5 ∙ 10^−4^ which enables to estimate the detection limit of our sensor as 1 ∙ 10^−6^.

**Figure 4:**
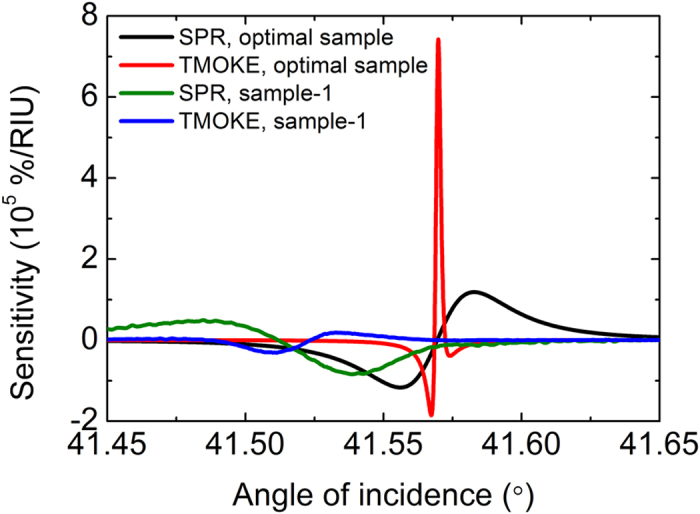
Comparison of SPR and TMOKE signal sensitivity. SPR and TMOKE signal sensitivity to the refractive index variation for the sample-1 (experimental data) and a sample with optimized parameters (theory). Detailed parameters of the structures are described in Methods.

Finally, we would like to note that an analysis shows that by further “fine tuning” of the parameters of the photonic crystal and metallic layers one could achieve even higher quality factor and sensitivity (Fig. 4). For the optimal Co layer thickness of 8 nm coated with 8 nm of Au, theoretical calculations predict the width of the SPR resonance as small as 0.03° (thus the quality factor is 1400) and the width of the TMOKE resonance as small as 0.002° (quality factor of 21000) (Supplementary Fig. S2). In the considered configuration the sensitivity of the SPR to the refractive index variation is 1.1 ∙ 10^5^%/RIU and TMOKE gives the sensitivity of 7.5 ∙ 10^5^%/RIU, which corresponds to the figure of merit of 2.1 ∙ 10^4^. These enormously high values demonstrate unambiguously a large room remaining for further progress in the discussed direction.

We presented a novel type of the surface plasmon sensor based on the magnetoplasmonic heterostructure. The most important feature of the sensor is an extremely high quality factor of the surface plasmon and magnetooptical resonances achieved by using the combination of the specially designed one dimensional photonic crystal and a pair of thin ferromagnetic cobalt and protective gold layers. The quality factor of the TMOKE resonances of such sensing structure (2000) is more than one order of magnitude higher than previously reported results. The magneto-optical resonance of our heterostructure has spectral width of 0.02° which is more than 10 times narrower than the values published before for the SPR long-range propagating sensors based on more standard optical reflection measurements. The experimentally obtained value of the magnetoplasmonic sensor figure of merit is at the level of the more complicated magnetoplasmonic nanosensors^34^, while our numerical simulations for the ideal structure show significantly higher results.

The other aspect of the obtained resonances is excitation of the ultra long-range magnetoplasmons with the estimated propagation length about 100 μm. This is of prime importance for modern magneto-optical non-reciprocal elements of the integrated optics that requires efficient magnetic field control of the propagating signals. Nowadays their capabilities are strongly limited by huge optical losses inserted by ferromagnetic metals. The heterostructure demonstrated here might take such hindrance away.

It is important to point out that such ultra-narrow resonances were achieved in the heterostructure which is relatively easy to fabricate. We can hope that further development of the structure, such as e.g. properly designed perforation of the metallic layers, could increase the quality factor and sensitivity even more. For example, structures with plasmonic Fabry-Perot resonances^35^ or with coupling between localized and propagating surface plasmon polaritons^36^ look rather promising in this respect. The proposed approach of plasmonic heterostructure SPR sharpening can be applied for various types of structures to get up to several orders of magnitude increase of their quality factors and sensitivities what is a vital problem for a wide range of SPR-sensor applications in biological and medical research.

## Methods

### Experimental setup

Experimental setup for sensor testing was constructed as follows. The laser diode Thorlabs QL7816S-B-L generates linearly polarized with the typical ratio of components 50:1 radiation with a spectral width around 0.3 nm and tunable in a spectral range of 780–798 nm via the change of the diode crystal temperature. The temperature of the laser box was stabilized with a precision of 0.01 K, and laser wavelength was controlled with a spectrometer Horiba HR-320. Laser radiation was collimated and focused onto sensor heterostructure surface by a combination of spherical and cylindrical lenses with focal distances 150 mm and 18 mm respectively in such a manner that a range of light incidence angles having a width around 3° is formed. The incident light is TM-polarized.

For the excitation of the SPR in a thin metal film of the magnetoplasmonic heterostructure we used the Kretschman configuration. Both the structure and the cylindrical lens were pasted on the corresponding planes of the prism made of BK7 glass and having a base angle of 42° using phase matching oil. The reflected radiation without any focusing was detected with a monochrome CMOS matrix of camera IDS UI-3360CP-M-GL having the 2048 × 1088 pixel screen with the sizes 11.264 mm × 5.984 mm and pixel size 5.5 μm thus realizing an angular interrogation scheme. The magnetoplasmonic heterostructure was placed inside the electromagnet with the interpolar gap 30 mm generating a magnetic field up to 30 mT in transversal Kerr effect configuration (orthogonal to the plane of the Fig. 1a). This field was strong enough to achieve the saturation of the sample magnetization. CMOS operation was handled via a home-written LabVIEW program that allowed us to control the electromagnet switching thus changing the polarity of the magnetic field, and to measure the real-time reflectance angular spectra as well as the TMOKE spectra.

The analyzed gas was delivered to a special cylindrical cell with gasket diameter of 12 mm, height of 2 mm and volume about 200 μl fixed on the metallic surface of plasmonic heterostructure. We used 3 gases as analytes: helium with 99.8% purity, air with 30% humidity, and the mixture of butane and propane gases with 70% of butane concentration that were blown in the cell at the atmosphere pressure and the room temperature of 21.6 °C. We used oil-free compressor for air supply, helium and butane were served from a gas cylinder at the pressure of 2 and 1.5 atmosphere, correspondingly. Between the measurements, the gas cell was blown with a large amount of air. The analyte gas flew through the cell in the volume of 400 ml at least to ensure that the cell is filled with it fully. The outlet hose was opened adjusting the pressure inside the cell to the level of atmosphere pressure preventing the structure from the mechanical deformations. The optical measurements were carried out 2 minutes after the cell was filled with the analyte gas in order to avoid any thermal effects. The variations of the gas compounds and corresponding variations of its refractive index were registered as the shift of reflection and TMOKE angular spectra. The penetration depth of the excited surface wave in gas as well as its propagation length were significantly smaller than the gas cell size so the impact of the cell walls was negligible.

### Magnetoplasmonic heterostructure

The magnetoplasmonic heterostructure was designed following the procedure outlined in^37^. The one dimensional photonic crystal, consisting of alternating Ta_2_O_5_/SiO_2_ layers deposited onto BK7 glass-made support plate plus the most external additional Ta_2_O_5_ layer has been prepared by magnetron sputtering. The widths of all layers, as well as the number of pairs of alternating layers, see Fig. 1b for concrete values, were selected to optimize the ultralong surface plasmon propagation conditions for the case when 8 nm thick Co layer plus some protective gold layer are deposited onto the photon crystal, and air (medium with the refractive index around 1.0003) is exploited as an external media^37^. Cobalt and gold layers were deposited onto finished photonic crystal by radio frequency magnetron sputtering. Actually measured resonance quality factors and TMOKE magnitudes in the fabricated samples are worse than the calculated ones due to deviations of the layer widths from the optimal values and some roughness of the Au and Co layers. Analysis of the sensor sensitivity deterioration under deviations of the layers thicknesses from the optimal values (Supplementary Fig. 3S) shows that although the requirements for the layer width are rather stringent, precision of the modern fabrication setups is enough to provide ultra-high quality factors in the real samples.

The detailed parameters of fabricated samples are as follows. The widths of SiO_2_ layers in photonic crystals of both samples are 164.7 nm, Ta_2_O_5_ layer 118.8 nm, additional Ta_2_O_5_ layer 107.5 nm. The width of Co layer is 10 nm, width of Au layer is 9 nm in sample-1 and the width of Co layer is 11 nm, width of Au layer is 18 nm in sample-2. The numerical simulations show that the widths of SiO_2_ layers equal to 164.0 nm, Ta_2_O_5_ layer - 119.4 nm, additional Ta_2_O_5_ layer - 108 nm, Co layer - 8 nm and Au layer - 8 nm are optimal for the observation of ultra-narrow SPR and TMOKE resonance.

The performed numerical simulations were based on rigorous coupled-wave analysis (RCWA) method. The dispersion was taken into account according to the published data for SiO_2_^38^, Ta_2_O_5_^39^, Au^40^ and Co^40^ and refined according to our experimental results so that at the operation wavelength of 786 nm permittivity of SiO_2_ was found to be 2.11, the permittivity of Ta_2_O_5_ was 4.47, the permittivity of Au was −24.14 + 5.17*i*, and the permittivity of Co was −9.22 + 33.00*i*. The gyration coefficient of Co layer magnetized up to its saturation value was found to be *g* = 0.51 + 1.17*i*.

## Additional Information

**How to cite this article**: Ignatyeva, D. O. *et al.* Magneto-optical plasmonic heterostructure with ultranarrow resonance for sensing applications. *Sci. Rep.* **6**, 28077; doi: 10.1038/srep28077 (2016).

## Supplementary Material

Supplementary Information
