# Supplementary material for: Magneto-optical plasmonic heterostructure with ultranarrow resonance for sensing applications
Source: Sci Rep. 2016 Jun 16;6:28077. doi: 10.1038/srep28077 (PMC4910117; doi:10.1038/srep28077)
Supplement: Supplementary Information [file srep28077-s1.pdf]

Supplementary information for
“Magneto-optical plasmonic heterostructure with ultranarrow resonance for sensing applications”

D.O. Ignatyeva, G.A. Knyazev, P.O. Kapralov, G. Dietler, S.K. Sekatskii, V.I. Belotelov

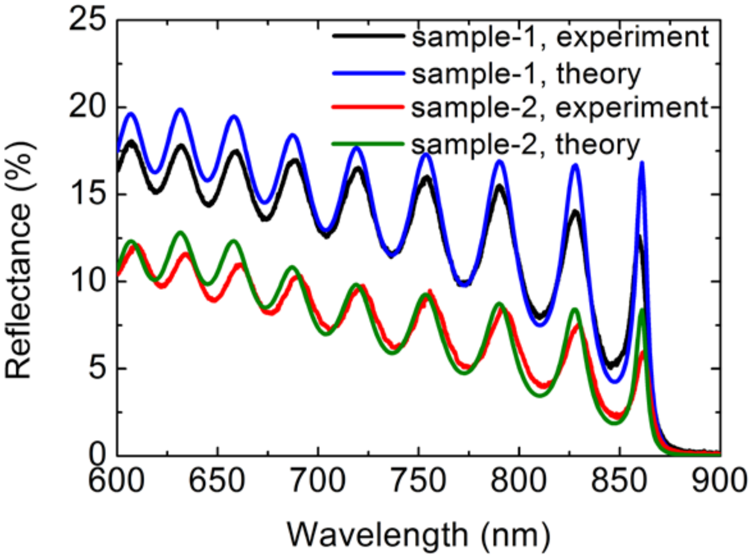

Figure 1S | Theoretical and experimental curves of the transmission spectra of the two samples corresponding to the normal incidence of light. Detailed parameters of the structures are described in Methods.

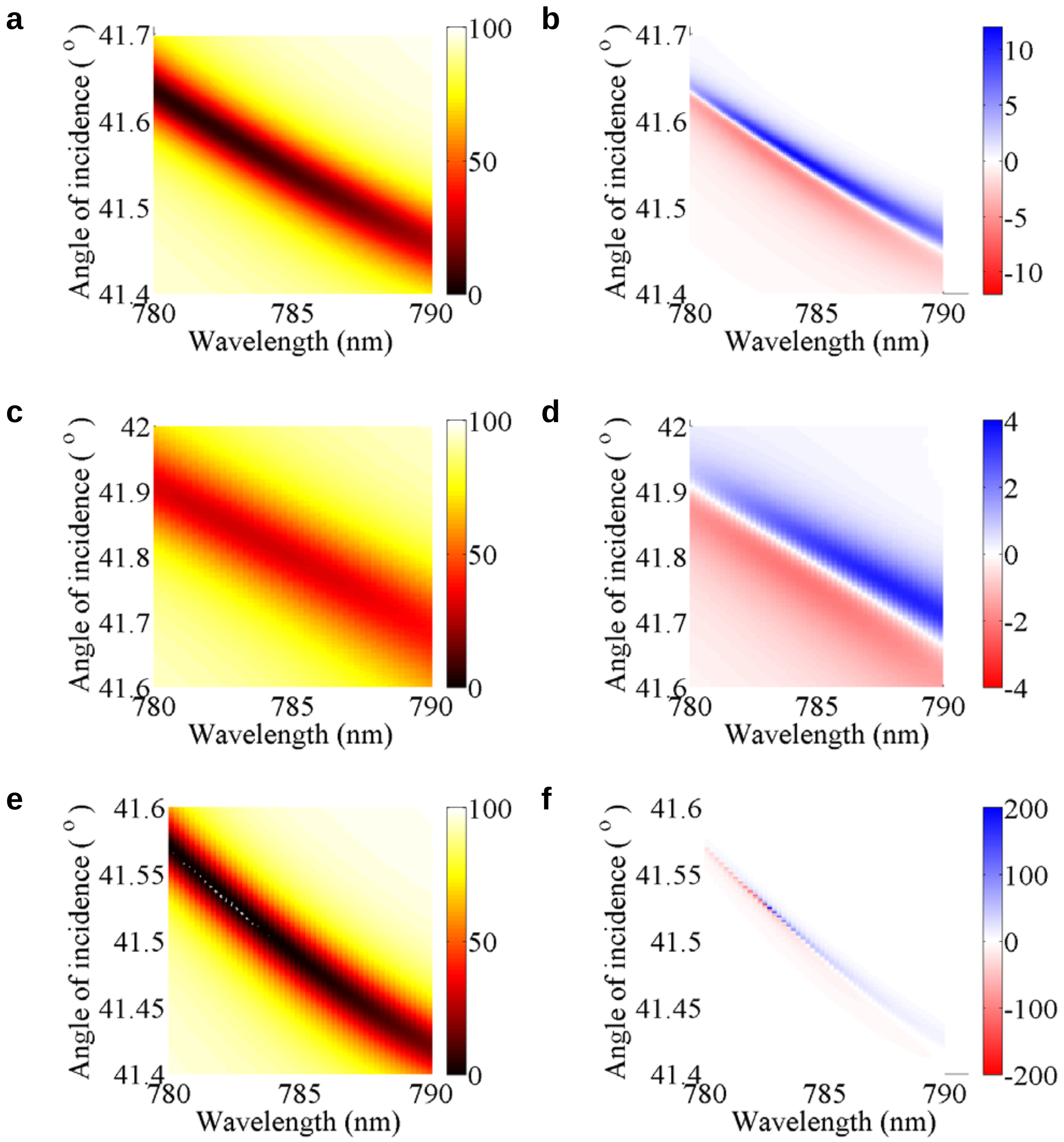

Figure 2S | The numerical calculation of (**a,c,e**) reflectance and (**b,d,f**) TMOKE for the sample-1 (**a,b**), the sample-2 (**c,d**) and optimized sample configuration (**e,f**). Detailed parameters of the structures are described in Methods.

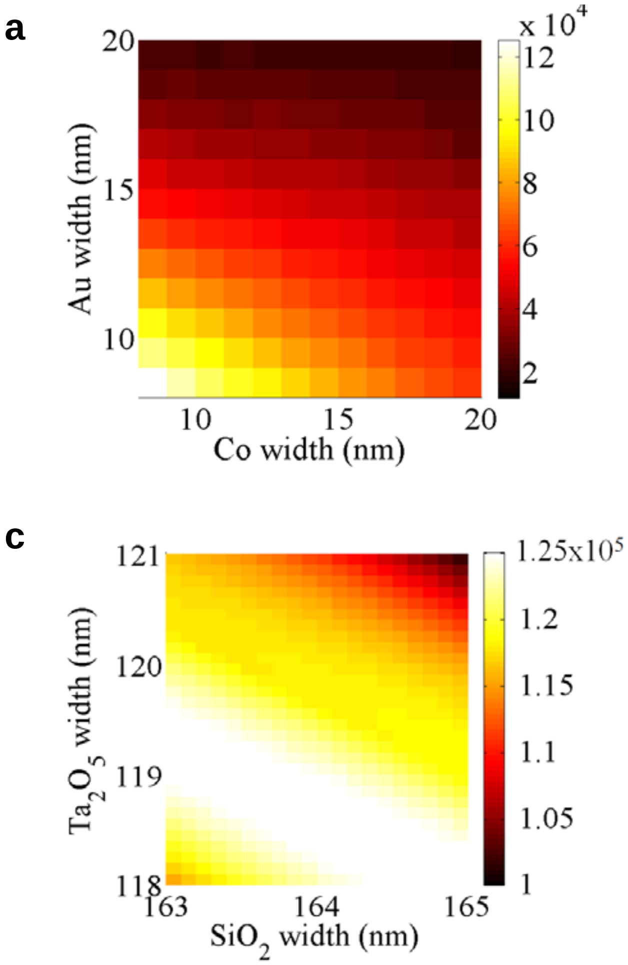

Figure 3S | The numerical calculation of (**a,c**) SPR and (**b,d**) TMOKE sensitivity for different width tolerance of Au, Co (**a,b**), and Ta_2O_5, SiO_2 (**c,d**) layers. See details in Methods.
